# The impact of the COVID-19 pandemic on transfers between long-term care and emergency departments across Alberta

**DOI:** 10.1186/s12873-023-00926-3

**Published:** 2024-01-07

**Authors:** Leanna Wyer, Yair Guterman, Vivian Ewa, Eddy Lang, Peter Faris, Jayna Holroyd-Leduc

**Affiliations:** 1 Alberta Health Services, Calgary, AB Canada; 2 Cumming School of Medicine, University of Calgary, Calgary, AB Canada

**Keywords:** COVID-19, Emergency department, Long-term care, Community paramedics

## Abstract

**Background:**

Long-term care (LTC) was overwhelmingly impacted by COVID-19 and unnecessary transfer to emergency departments (ED) can have negative health outcomes. This study aimed to explore how the COVID-19 pandemic impacted LTC to ED transfers and hospitalizations, utilization of community paramedics and facilitated conversations between LTC and ED physicians during the first four waves of the pandemic in Alberta, Canada.

**Methods:**

In this retrospective population-based study, administrative databases were linked to identify episodes of care for LTC residents who resided in facilities in Alberta, Canada. This study included data from January 1, 2018 to December 31, 2021 to capture outcomes prior to the onset of the pandemic and across the first four waves. Individuals were included if they visited an emergency department, received care from a community paramedic or whose care involved a facilitated conversation between LTC and ED physicians during this time period.

**Results:**

Transfers to ED and hospitalizations from LTC have been gradually declining since 2018 with a sharp decline seen during wave 1 of the pandemic that was greatest in the lowest-priority triage classification (CTAS 5). Community paramedic visits were highest during the first two waves of the pandemic before declining in subsequent waves; facilitated calls between LTC and ED physicians increased during the waves.

**Conclusions:**

There was a reduction in number of transfers from LTC to EDs and in hospitalizations during the first four waves of the pandemic. This was supported by increased conversations between LTC and ED physicians, but was not associated with increased community paramedic visits. Additional work is needed to explore how programs such as community paramedics and facilitated conversations between LTC and ED providers can help to reduce unnecessary transfers to hospital.

## Background

The COVID-19 pandemic has had an unprecedented impact on long term care (LTC) residents in Canada [1]. On March 15, 2020, a state of public health emergency was declared in Alberta whereby elective surgeries were postponed and healthcare visits were primarily shifted to a virtual format to limit exposure. In addition, emergency department (ED) visits and hospital admissions declined as individuals were afraid of exposing themselves to the coronavirus [2]. LTC facilities struggled to contain viral spread, leading to extensive outbreaks. The Canadian LTC sector was particularly negatively impacted [3] compared to other countries, with estimates suggesting that nearly 80% of COVID-19 deaths in the first wave took place in LTC [4, 5].

Alberta is in a unique position in that it has a single provincial health authority that serves a population of 4.4 million, including a close working relationship with LTC operators across the province [6]. This has helped to facilitate a more coordinated response to the pandemic, whereas other provinces struggled with distribution of personal protective equipment to LTC and experienced testing supply shortages [1]. The importance of the relationship between LTC and acute care has been previously highlighted both through the implementation of a collaborative care program in Ontario [7] and by comparing the effect of the pandemic in British Columbia and Ontario [8].

A report created by the Canadian Institute for Health Information stated that LTC residents received less medical care in the first wave of the pandemic, partly attributed to less transfers to hospital [9]. However, the change in health care resource utilization observed during the pandemic does not necessarily mean that LTC residents did not receive needed acute care. There are often options to provide needed medical care within LTC facilities, and there can also be negative outcomes associated with hospital transfers among LTC residents living with frailty. Choosing Wisely Canada guidelines recommend that residents not be transferred to hospital unless their care needs cannot be address at the LTC facility [10]. This is a recommendation that existed prior to the pandemic and its importance was amplified as LTC facilities in Canada were significantly impacted by COVID-19 [3]. As such, it is important to not only investigate how the pandemic impacted transfers to ED but also how community-based resources were utilized to showcase alternative options available to support changes in health status of LTC residents. Previous work has focused primarily on the early days of the pandemic [11] and as such, more work is needed to explore the experience in LTC beyond 2020. We aimed to explore how the COVID-19 pandemic impacted LTC to ED transfers and hospitalizations across Alberta, Canada during the first four waves of the pandemic, including looking at changes based on level of acuity. We also explored the impact on utilization of community paramedics within LTC and the role of facilitated LTC to ED physician conversations to address how care was provided to LTC residents outside of acute care.

## Methods

### Study design and setting

We conducted a retrospective population-based study to explore healthcare resource utilization outcomes among LTC residents in the province of Alberta, Canada across the first four waves of the pandemic. Alberta has 186 LTC facilities with 15, 762 beds (as of February 28, 2021) [12] and Alberta Health Services has 106 acute care hospitals [13]. Outcomes were compared across wave 1 (March 5, 2020– May 3, 2020), wave 2 (November 1, 2020– February 1, 2021), wave 3 (March 15, 2021– June 1, 2021), and wave 4 (September 1, 2021– November 15, 2021) [14]. Additionally, January 6, 2021 represents when first doses of vaccines were administered to LTC residents in Alberta.

### Participants

The study population included any individuals residing in a LTC facility in Alberta, Canada who visited an emergency department, received care from a community paramedic, or whose care involved RAAPID or OLMC facilitated provider calls during the study period of January 1, 2018 and December 31, 2021, where available. RAAPID (Referral, Access, Advice, Placement, Information & Destination) is a provincial call centre that links a physician from a referring facility (e.g. LTC) to a physician in an accepting facility (e.g. hospital) or emergency department. OLMC (Online Medical Consultation) links paramedics in the field with ED physicians.

### Data sources

We linked several province-wide administrative datasets using individual personal health numbers to identify episodes of care. These datasets included emergency department visits (National Ambulatory Care Reporting System), long term care facility data (Alberta Continuing Care Information System), hospital admissions (Discharge Abstract Database), community paramedicine data (Siren, Computer Aided Dispatch, Integrated Reporting Information System), RAAPID facilitated calls (Clarity, Coordination and Referral Information System) and OLMC data (Shock Trauma Air Rescue Service (STARs) Computer Aided Dispatch).

### Data analysis

Total number of transfers to ED, hospitalizations, community paramedic visits, as well as calls to RAAPID and OLMC were calculated monthly across the study period. Transfers to ED, stratified by sex and acuity, and hospitalizations during each wave were also calculated. Data is presented from 2019 to serve as a pre-pandemic comparison; the percent change was calculated to quantify the decline seen in ED transfers and hospitalizations, considering patient acuity.

### Measurements and outcomes

The outcomes of interest included transfers from LTC to ED, hospital admissions, and utilization of community paramedics, RAAPID facilitated physician-to-physician calls and OLMC facilitated paramedic-to-ED physician calls. ED visits were stratified by the Canadian Triage Acuity Scale (CTAS) score within the available administrative dataset, as a Nationally standardized way to indicate patient acuity [15]. CTAS 1 represents the highest acuity and 5 the lowest acuity. CTAS scores of 4–5 are typically felt not to need treatment within an ED. Community paramedic data only included LTC residents who were treated and not transported. Cases involving community paramedics where care was refused, no patient was found, backup was provided or the patient was transported by another provider, were not included. Both OLMC facilitated paramedic-to-ED physician and RAAPID facilitated LTC physician-to-ED physician call data included any call regardless of the disposition.

### Ethics approval

This study was approved by the University of Calgary Conjoint Health research Ethics Board (REB21-0147) in accordance with the Tri-council Policy Statement (TCPS2 2022), Ethical Conduct for Research Involving Humans. Informed consent was waived as this study utilized non-identifiable data from administrative data sources.

## Results

### Transfers to ED and hospital admissions

**Fig. 1:**
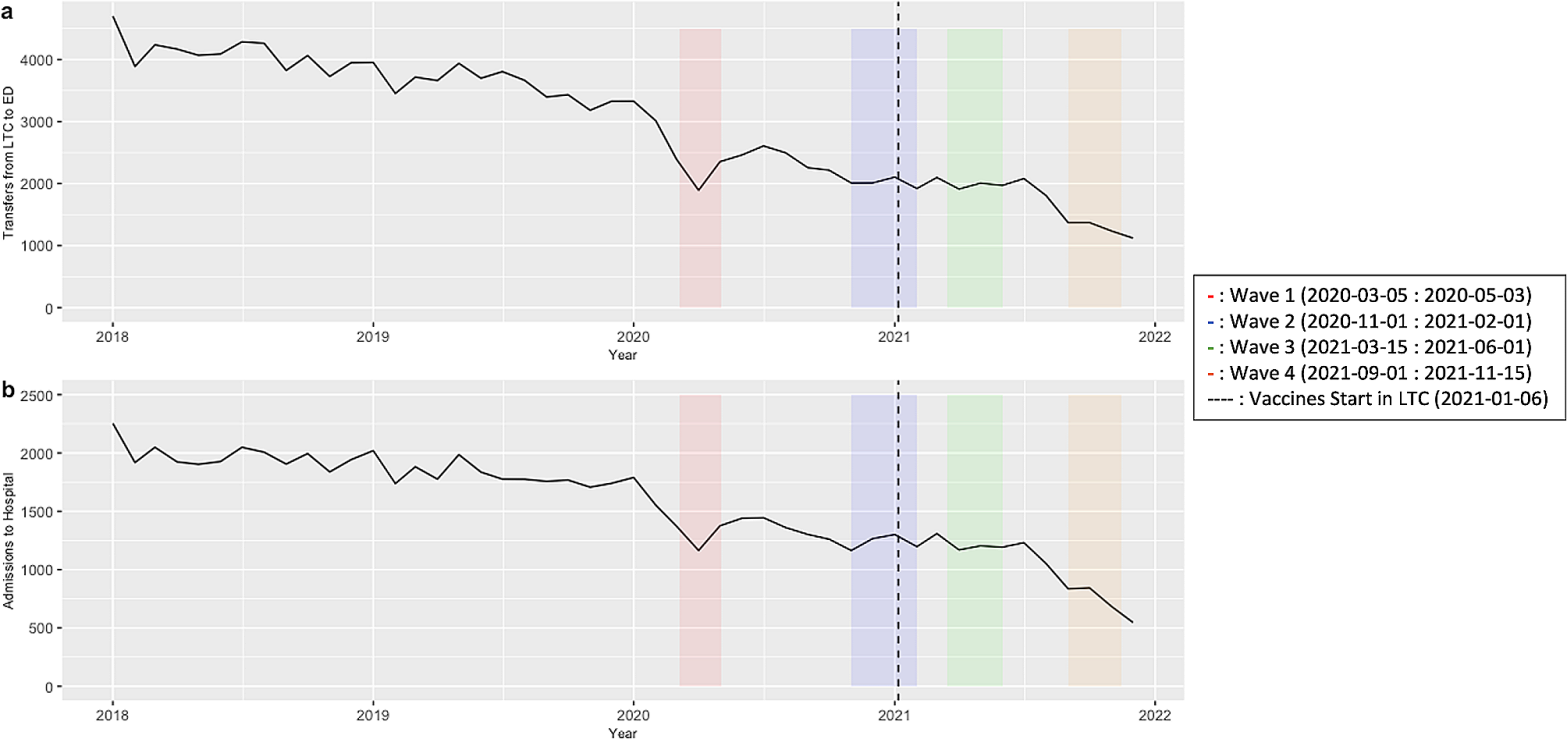
Q1(A) Transfers from Long-term Care (LTC) to Emergency Departments (ED) and (B) admissions to hospital from January 1, 2018 until December 31, 2021. Colored bars indicate the waves of the pandemic and the dashed line indicates when vaccines were first administered to LTC residents

Since 2018, both transfers from LTC to ED and admissions to hospital have been gradually declining (Fig. 1). However, for both outcomes a sharp decline was observed during the first wave of the COVID-19 pandemic. The number of transfers between waves 1 and 3 remained relatively stable, before declining further around wave 4. This same trend was also seen with hospital admissions. Compared to 2019, during waves 1 and 2 there was a 44% and 38% reduction in ED visits and a 33% and 29% reduction in hospital admissions. The first dose of vaccine was offered to this population near the end of the second wave, with administration of the second dose occurring near the start of wave 3. The characteristics of LTC residents being transferred between March 2019 (pre-pandemic) and November 2021 (end of wave 4) are presented in Table 1.

**Table 1:** Characteristics of LTC residents

		2019–2020		2020–2021			
		Mar 5/19 - May 3/19	Nov 1/19 - Feb 1/20	Mar 5/20 - May 3/20 (Wave 1)	Nov 1/20 - Feb 1/21 (Wave 2)	Mar 15/21 - Jun 1/21 (Wave 3)	Sept 1/21 - Nov 15/21 (Wave 4)
Total ED visits		7296	9945	4079	6192	5145	3384
Sex	Male	3271	4616	1913	3005	2473	1547
	Female	4025	5329	2166	3187	2672	1837
Mean Age		79	79	78	79	79	78
Number of Hospital Admissions		3652	5298	2433	3772	3120	2036
CTAS scores	1	190	281	165	298	227	181
	2	1566	2236	944	1628	1336	953
	3	3444	4635	1957	2863	2462	1621
	4	1505	1975	735	1020	804	451
	5	361	517	156	203	188	107

ED: emergency department; CTAS: Canadian Triage Acuity Scale

### Patient acuity

**Fig. 2:**
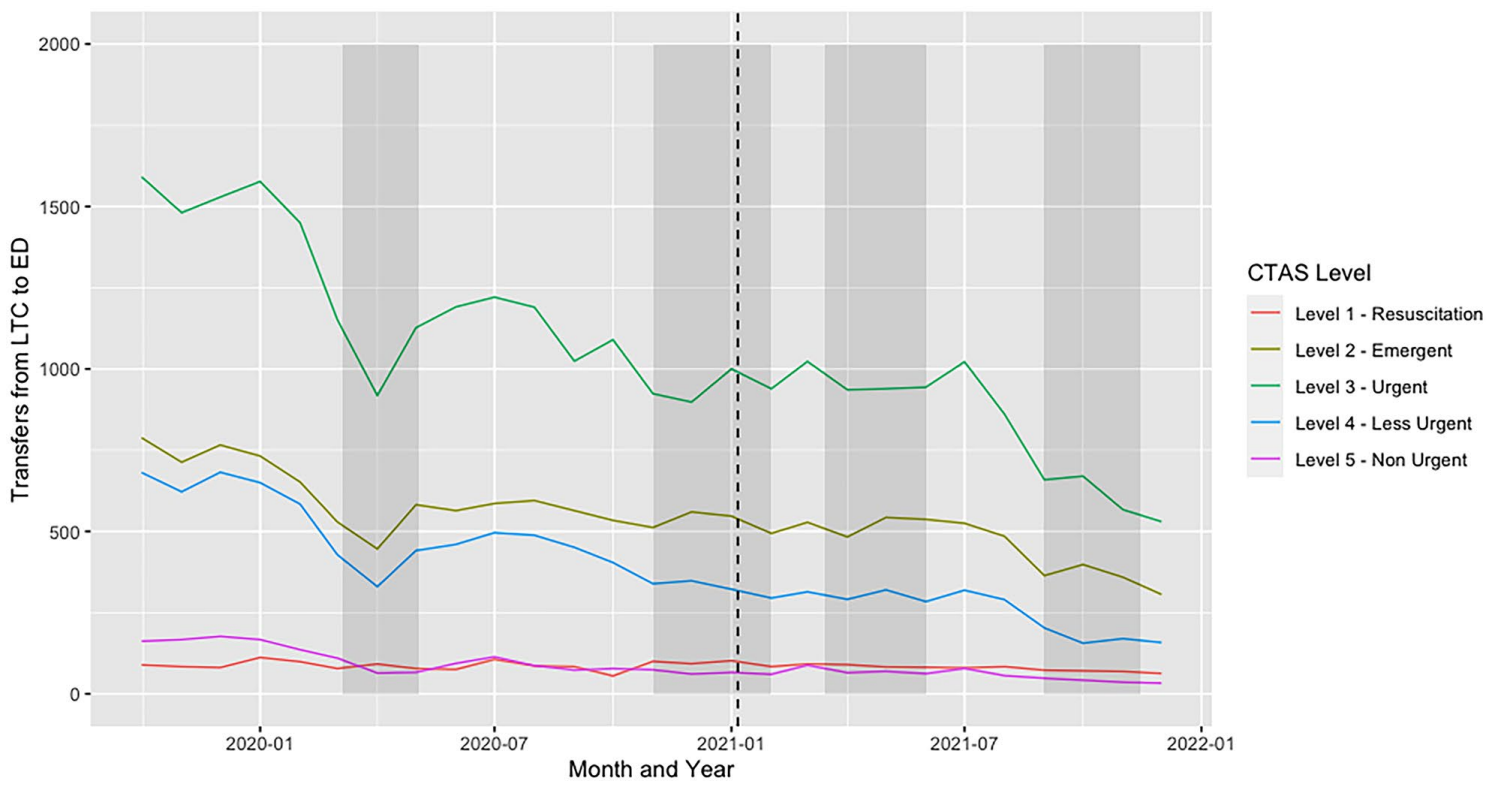
Transfers from long-term care (LTC) to emergency departments (ED) stratified by Canadian Triage Acuity Scale (5 = lowest, 1 = highest) from November 1, 2019 until December 31, 2021. Shaded bars indicate waves of the pandemic. The dashed line represents when vaccines were first administered to LTC residents

Over the course of the pandemic, LTC residents most frequently presented to the ED with an acuity of CTAS 3 followed by CTAS 2 (Fig. 2). The proportion of calls related to the highest-priority (CTAS 1) and lowest-priority (CTAS 5) triage classification remained low throughout. All triage classifications saw a sharp decline during wave 1, in line with the large reduction in transfers to ED overall (Fig. 2). However, the reduction was greatest (57%) in the lowest-priority triage classification (CTAS 5) compared to only a 13% reduction for CTAS 1.

### Community paramedic visits and facilitated calls between healthcare providers

**Fig. 3:**
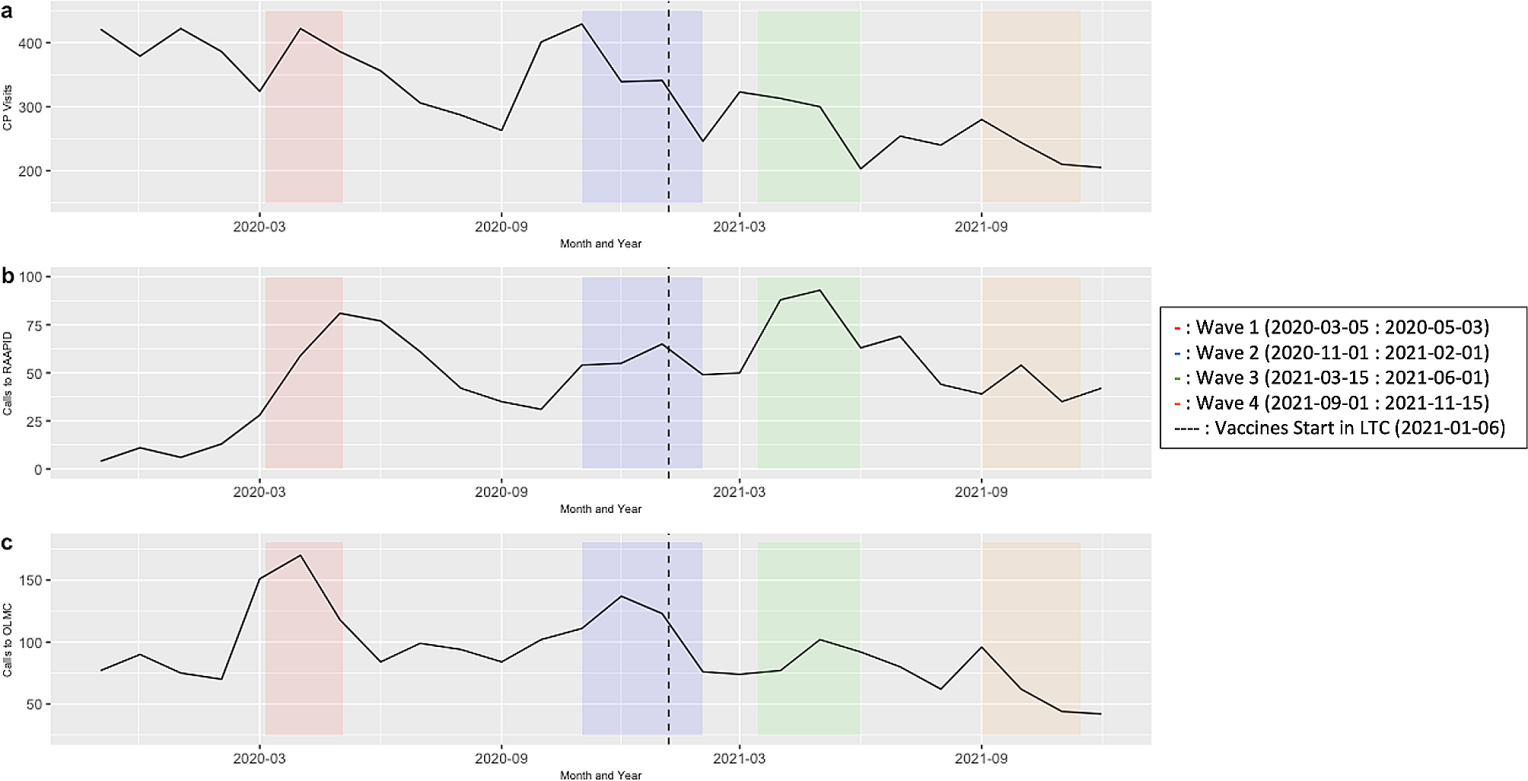
(A) Community paramedic (CP) visits to long-term care (LTC) facilities where residents were treated and not transported, (B) RAAPID facilitated LTC physician-to-ED physician calls regardless of disposition and (C) OLMC facilitated paramedic-to-ED physician calls at LTC facilities from November 1, 2019 until December 31, 2021. Colored bars indicate the waves of the pandemic and the dashed line indicates when vaccines were first administered to LTC residents

Despite an initial increase in visits from community paramedics during the first two waves of the pandemic, subsequent waves were associated with a reduction in visits to LTC (Fig. 3). During the first wave of the pandemic RAAPID facilitated LTC physician-to-ED physician calls saw a large increase, reaching their peak during wave 3 (Fig. 3). Overall, these facilitated calls were higher throughout the pandemic when compared to the time period prior. OLMC facilitated paramedic-to-ED physician calls reached a peak during the first wave, with smaller peaks being seen in waves 2 and 3 before there was a reduction in call volume throughout wave 4 (Fig. 3).

## Discussion

The COVID-19 pandemic has markedly impacted individuals living in LTC, a population who is also prone to negative health outcomes associated with unnecessary transfer to ED [16, 17]. Limiting resident exposure to COVID-19 in both LTC and hospital settings, while also ensuring appropriate medical care was delivered in the most appropriate location, was a challenge throughout the pandemic. Similar to other studies in Canada [2, 18], the United States [19, 20] and the United Kingdom [21], we found that the first four waves of the pandemic were associated with reductions in LTC resident transfers to ED. Of note, the smallest and largest decreases in ED visits were those classified as CTAS 1 and CTAS 5, respectively, suggesting that individuals with the highest acuity were still being transferred and those with less urgent changes in health status could be addressed outside of acute care. We also found an associated reduction in admissions to hospitals and an increase in facilitated LTC physician to ED physician conversations.

Community paramedics were not clearly utilized within LTC as an alternative to ED transfers during the pandemic. Despite not finding an increase in community paramedic visits throughout the pandemic, this cannot negate the extreme value that community paramedics provide to this population [22, 23]. The need for community paramedics during the pandemic may have been impacted by the fact that supports were put into Alberta LTC facilities to enable staff to provide additional medical care to residents, in an attempt to limit unnecessary interaction with outside healthcare providers and thus limit exposure to the virus. The reduction in both community paramedic visits and ED transfers observed during the pandemic is likely explained in part by this change within LTC. However, fear around contracting COVID-19 in hospital [24], worry about being a burden to the healthcare system and concern about preserving hospital capacity [25] are also potential explanations for the decline in ED transfers. The high prevalence of facility outbreaks in both LTC and acute care, along with known negative health outcomes associated with ED transfers among LTC residents, meant the decision to transfer residents to acute care during the pandemic was complex [26].

In the early days of the pandemic, LTC sites in Alberta were instructed by public health to utilize RAAPID facilitated physician-to-physician calls prior to transferring LTC residents with COVID to an ED. In addition, the desire to minimize resident transfers, LTC physicians could utilize Specialist Link [27] as a means by which to receive telephone support from other specialist physicians. RAAPID facilitated physician-to-physician calls in Alberta [28] and Rapid Access to Consultative Expertise (RACE) [29] in British Columbia have shown that telephone consultations between healthcare providers can lead to reductions in transfers to ED for other populations. Overall, the involvement of these provincial programs appeared to support residents remaining at their LTC facility when safe and appropriate to do so, as supported by the greater decrease seen in ED transfers for less acute non-urgent health issues. Additional work completed in LTC both before [30] and after [7, 31] the onset of the pandemic has further emphasized how collaboration between LTC and acute care can help to reduce unnecessary transfers to hospital.

LTC residents were considered high priority for vaccination when they became available, and LTC residents in Canada began receiving vaccines in January of 2021. Vaccination has been shown to reduce ED visits and hospital admissions for those ≥ 65 years [32] and all-cause hospitalization regardless of age in both Alberta and Ontario [33]. This aligns with the trends we saw in both ED visits and hospital admissions when comparing the time pre and post vaccination among LTC residents.

This study was able to examine healthcare resource utilization among LTC residents throughout multiple waves of the pandemic and across a large healthcare system. We were able to explore impact on community resources, in addition to the more commonly evaluated outcomes of ED transfers and hospitalization. However, this is a descriptive study exploring only changes in healthcare resource utilization. We did not look at impacts the pandemic had on LTC resident outcomes such as COVID-related morbidity or mortality. Future work is needed to provide a more comprehensive explanation for the reasons behind the changes in healthcare resource utilization seen, to correlate these to LTC resident outcomes and to determine if these changes within the healthcare system persist over time.

## Conclusions

During the first four waves of the pandemic, we observed a reduction in the number of LTC residents transferred to EDs and admitted to hospital in Alberta, particularly among residents experiencing lower acuity changes in health. This reduction in transfers was supported by an increase in facilitated LTC physician to ED physician calls. However, community paramedics were not clearly utilized within LTC as an alternative to ED transfers during the pandemic. Given the immense pressures currently impacting EDs and emergency medical services in Canada, along with the higher risk of negative health outcomes for LTC residents when transferred to hospital, solutions are needed to help alleviate these systemic issues. Access to additional medical care resources onsite, including through community paramedic consultations, could help to provide LTC staff with needed supports to appropriately address acute medical issues onsite, thus reducing transfers to ED. When transfers are necessary, facilitated physician-to-physician telephone conversations can help to improve communication between LTC and ED providers, thus better ensuring optimal care for LTC residents living with frailty and complex comorbidities.

Additional work is needed to look at how programs such as community paramedicine and telephone consultation services can help to reduce unnecessary transfers from LTC to ED, improve communication between healthcare providers and avoid healthcare costs, all while focusing on providing LTC residents with the right care at the right location.

## Data Availability

All datasets used and/or analysed during this study are not publicly available due to the personal health information they contain.

## Abbreviations

LTC: Long-term care
ED: emergency department
CTAS: Canadian Triage Acuity Scale
RAAPID: Referral, Access, Advice, Placement, Information & Destination
OLMC: Online Medical Consultation
STARs: Shock Trauma Air Rescue Service
CP: Community paramedic
RACE: Rapid Access to Consultative Expertise
